# Data on molecular identification, phylogeny and *in vitro* characterization of bacteria isolated from maize rhizosphere in Cameroon

**DOI:** 10.1016/j.dib.2018.06.003

**Published:** 2018-06-11

**Authors:** Gylaine Vanissa Tchuisseu Tchakounté, Beatrice Berger, Sascha Patz, Henri Fankem, Silke Ruppel

**Affiliations:** a Leibniz Institute of Vegetable and Ornamental Crops Grossbeeren/ Erfurt e.V., Theodor- Echtermeyer-Weg 1, 14979 Grossbeeren, Germany; b Institute for National and International Plant Health, Julius Kuehn-Institute – Federal Research Centre for Cultivated Plants, Messeweg 11/12, 38104 Braunschweig, Germany; c Algorithms in Bioinformatics, Center for Bioinformatics, University of Tuebingen, Sand 14, 72076 Tuebingen, Germany; d Department of Plant Biology, Faculty of Sciences, University of Douala, P.O.Box: 24157, Douala, Cameroon; e Faculty of Life sciences Humboldt-University of Berlin, Invalidenstraße 42, 10115 Berlin, Germany

**Keywords:** Bacteria, Maize rhizosphere, Taxonomical affiliation, Functional traits, Growth promotion

## Abstract

Bacteria, which establish positive interactions with plant roots, play a key role in agricultural environments and are promising for their potential use in sustainable agriculture. Many of these mutualistic bacteria provide benefits to plant hosts by facilitating soil mineral nutrient uptake, protecting plants from biotic and abiotic stresses and producing substances that promote growth. The dataset presented here, is related to the publication entitled “Community structure and plant growth-promoting potential of cultivable bacteria isolated from Cameroon soil” (Tchuisseu et al., 2018) [1]. The data provide an extended analysis of the occurrence, taxonomical affiliation and functional traits of bacterial groups isolated from the rhizosphere of maize in Cameroon at different taxonomical levels, using a combination of molecular/bioinformatics tools and *in vitro* studies. Bacteria were isolated from maize rhizosphere soil. Isolated bacteria were identified using the 16s rRNA gene sequencing and phylogenetic analyses. All strains were characterized for their potential of salinity tolerance and growth promotion (phosphate solubilization, *nifH* gene presence and siderophore production) in order to select efficient bacterial strains for designing biological fertilizer exploitable for agriculture under specific stress conditions of the country. The data will be valuable for further studies on plant associated bacteria in Cameroon, which are still largely unexplored.

**Specifications Table**

**Table:** 

Subject area	Biology
More specific subject area	Microbiology, Biotechnology
Type of data	Table, Figure
How data was acquired	Isolation of bacteria from rhizosphere soil of maize field.
	Molecular identification of isolates and phylogenetic analyses.
	*In vitro* screening of bacterial group based on their different functional traits.
Data format	Raw, Analyzed
Experimental factors	DNA extraction of all isolates, determination of DNA concentration and quality check. 16S rDNA sequences were analyzed an edited using NCBI and Ribosomal Database Project (RDP), assembled using Muscle and the phylogenetic analysis was proceeded using Mega 7 version 7.0.21.
	Biochemical test to evaluate all bacterial strains for their salt tolerance, phosphate solubilization, *nifH* gene possession and siderophore production potential.
Experimental features	Isolation of bacterial isolates, followed by the identification using the 16S rRNA gene sequences, and then, the *in vitro* characterization for the different functional traits tested.
Data source location	Soil samples were collected in Ngaoundal locality (6° 30′′ North, 13° 16′′ East), Cameroon.
Data accessibility	Data are presented in this article.

**Value of the data**

• Like in many sub-Sahara African countries, studies on plant growth-promoting bacteria (PGPB) are still largely untapped in Cameroon. This data provides the first collection of bacteria associated with maize in Cameroon and therefore is valuable for further studies of microbial communities associated with plants in the country.
• The different traits tested give insight into the functional difference between bacterial groups found in the maize rhizosphere and can be useful to understand the ecological role of newly isolated bacteria in their specific environment.
• The knowledge about the bacterial groups and their functional traits may contribute to improve management practices regarding plant resistance to salinity and plant nutrition.
• This data may contribute in promoting microbial bio- fertilizers based on native rhizobacteria in sub-Saharan Africa and enhancing sustainable crop production systems.

## 1 Data

**Fig. 1:**
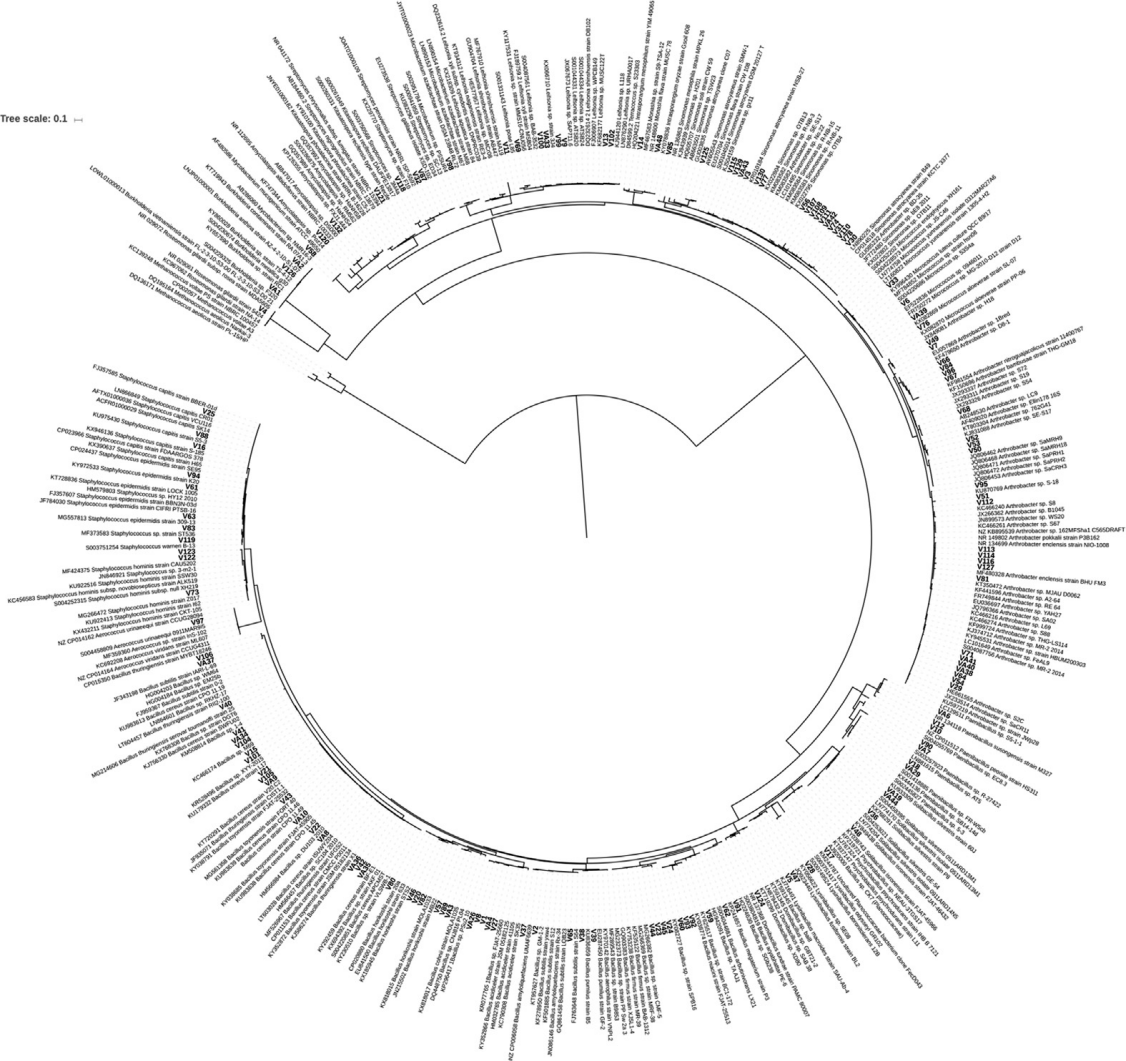
Phylogenetic tree based on 16S rDNA sequences revealing phylogenetic classification of the 143 isolates. The Maximum Likelihood tree was structured using the Tamura 3-parameter model and the neighbor joining method. *Methanococcus* ssp. was used as outgroup. The isolates between sequences are represented in bold.

The study of plant-associated microorganisms is of great importance for biotechnological applications, for example, resistance to abiotic and biotic stresses, plant growth-promotion, or isolation of active compounds [2]. The benefit that PGP bacteria exert to plant growth and yield is well known. However, the growth-promoting effect depends mainly on native biotic and abiotic factors including bacterial species and the soil types [1]. Therefore, knowledge about the native bacterial populations, their identification and their implications for plant physiology, is required for improving management practices regarding plant nutrition and resistance to abiotic stresses [3]. The dataset of this article provides information on the community and functional difference of different groups of bacteria isolated from maize rhizosphere soil in Cameroon. Phylogenetic analysis was used to cluster isolates to their closely related species (Fig. 1) where, the branch lengths displayed represent substitutions per site. Table 1 presents the ability of different bacterial groups to tolerate increasing concentration of sodium chloride (NaCl) at genus, family and phylum level. Table 2 shows the characterization of bacterial strains to solubilize seven different compounds of inorganic phosphate: tricalcium-phosphate, hydroxyapatite, Malian rock phosphate (RP), Cameroonian RP, Algerian RP, Mexican RP, Moroccan RP at genus, family and phylum level. Table 3 shows the ability of bacterial groups to possess *nifH* gene, responsible for nitrogen fixation and to produce siderophore at genus, family and phylum level.

**Table 1:** Occurrence and characterization of bacterial strains to tolerate different concentration of salt at genus, family and phylum level.

**Characterization level**		**Number of isolates**	**2% NaCl**	**4% NaCl**	**6% NaCl**	**8% NaCl**
**Genera**	***Aerococcus***	1	1	1	1	1
	***Amycolatopsis***	2	1	1	1	0
	***Arthrobacter***	25	24	15	5	1
	***Bacillus***	45	38	31	22	10
	***Burkholderia***	3	2	0	0	0
	***Domibacillus***	1	0	0	0	0
	***Kitasatospora***	1	0	0	0	0
	***Leifsonia***	8	7	1	0	0
	***Lysinibacillus***	3	3	2	1	0
	***Microbacterium***	1	1	0	0	0
	***Micrococcus***	4	4	4	4	2
	***Mycobacterium***	1	1	1	0	0
	***Paenibacillus***	7	7	4	1	1
	***Roseomonas***	1	0	0	0	0
	***Sinomonas***	19	19	18	1	0
	***Solibacillus***	4	4	4	1	0
	***Staphylococcus***	11	11	8	8	8
	***Streptomyces***	3	1	1	1	1
	***Unclassified Intrasporangiaceae***	2	1	0	0	0
	***Unclassified Planococcaceae***	1	1	1	1	0
Total	**20**					
**Families**	***Acetobacteraceae***	1	0	0	0	0
	***Aerococcaceae***	1	1	1	1	1
	***Bacillaceae***	49	41	33	23	10
	***Burkholderiaceae***	3	2	0	0	0
	***Intrasporangiaceae***	2	1	0	0	0
	***Microbacteriaceae***	9	8	1	0	0
	***Micrococcaceae***	48	47	37	10	3
	***Mycobacteriaceae***	1	1	1	0	0
	***Paenibacillaceae***	7	7	4	1	1
	***Planococcaceae***	5	5	5	2	0
	***Pseudonocardiaceae***	2	1	1	1	0
	***Staphylococcaceae***	11	11	8	8	8
	***Streptomycetaceae***	4	1	1	1	1
Total	**13**					
**Phyla**	***Actinobacteria***	66	59	41	12	4
	***Firmicutes***	73	65	51	35	20
	***Proteobacteria***	4	2	0	0	0
Total	**3**	**143**	**126**	**92**	**47**	**24**

**Table 2:** Occurrence and characterization of bacterial strains to solubilize different types of inorganic phosphate source at genus, family and phylum level.

**Characterization level**		**Number of isolates**	**Chemical inorganic phosphate**		**Rock phosphate (RP)**					**Total P solubizing isolates**
			**Tricalcium Phosphate**	**Hydroxyapatite**	**Malian RP**	**Cameroonian RP**	**Algerian RP**	**Mexican RP**	**Moroccan RP**	
**Genera**	***Aerococcus***	1	0	0	0	0	0	0	0	0
	***Amycolatopsis***	2	1	1	1	0	0	1	0	1
	***Arthrobacter***	25	19	14	18	10	15	12	2	19
	***Bacillus***	45	18	14	17	13	11	12	2	20
	***Burkholderia***	3	0	0	0	0	0	0	0	0
	***Domibacillus***	1	0	0	0	0	0	0	0	0
	***Kitasatospora***	1	0	0	0	1	0	0	0	1
	***Leifsonia***	8	3	2	3	2	1	1	0	3
	***Lysinibacillus***	3	0	0	0	0	0	0	0	0
	***Microbacterium***	1	1	1	1	1	1	1	0	1
	***Micrococcus***	4	3	2	3	3	2	2	0	3
	***Mycobacterium***	1	0	0	0	0	0	0	0	0
	***Paenibacillus***	7	5	5	3	2	4	4	0	7
	***Roseomonas***	1	0	0	1	1	0	0	0	1
	***Sinomonas***	19	12	1	11	6	3	2	0	12
	***Solibacillus***	4	0	0	0	0	0	0	0	0
	***Staphylococcus***	11	3	1	3	2	1	1	0	3
	***Streptomyces***	3	1	1	1	1	1	0	0	1
	***Unclassified Intrasporangiaceae***	2	0	0	0	0	0	0	0	0
	***Unclassified Planococcaceae***	1	0	0	0	0	0	0	0	0
Total	**20**									
**Families**	***Acetobacteraceae***	1	0	0	1	1	0	0	0	1
	***Aerococcaceae***	1	0	0	0	0	0	0	0	0
	***Bacillaceae***	49	18	14	17	13	11	12	2	20
	***Burkholderiaceae***	3	0	0	0	0	0	0	0	0
	***Intrasporangiaceae***	2	0	0	0	0	0	0	0	0
	***Microbacteriaceae***	9	4	3	4	3	2	2	0	4
	***Micrococcaceae***	48	34	17	32	19	20	16	2	34
	***Mycobacteriaceae***	1	0	0	0	0	0	0	0	0
	***Paenibacillaceae***	7	5	5	3	2	4	4	0	7
	***Planococcaceae***	5	0	0	0	0	0	0	0	0
	***Pseudonocardiaceae***	2	1	1	1	0	0	1	0	1
	***Staphylococcaceae***	11	3	1	3	2	1	1	0	3
	***Streptomycetaceae***	4	1	1	1	2	1	0	0	2
Total	**13**									
**Phyla**	***Actinobacteria***	66	40	22	38	24	23	19	2	41
	***Firmicutes***	73	26	20	23	17	16	17	2	30
	***Proteobacteria***	4	0	0	1	1	0	0	0	1
Total	**3**	**143**	**66**	**42**	**62**	**42**	**39**	**36**	**4**	**72**

**Table 3:** Occurrence and characterization of bacterial strains for *nifH* gene presence and siderophore production at genus, family and phylum level.

**Characterization level**		**Number of isolates**	***nifH*****gene**	**Siderophore production**
**Genera**	***Aerococcus***	1	0	0
	***Amycolatopsis***	2	0	0
	***Arthrobacter***	25	7	2
	***Bacillus***	45	3	13
	***Burkholderia***	3	0	1
	***Domibacillus***	1	0	0
	***Kitasatospora***	1	0	1
	***Leifsonia***	8	1	1
	***Lysinibacillus***	3	1	0
	***Microbacterium***	1	0	0
	***Micrococcus***	4	0	1
	***Mycobacterium***	1	0	1
	***Paenibacillus***	7	2	1
	***Roseomonas***	1	0	1
	***Sinomonas***	19	0	0
	***Solibacillus***	4	0	0
	***Staphylococcus***	11	1	5
	***Streptomyces***	3	0	0
	***Unclassified Intrasporangiaceae***	2	0	1
	***Unclassified Planococcaceae***	1	0	0
**Total**	**20**			
**Families**	***Acetobacteraceae***	1	0	1
	***Aerococcaceae***	1	0	0
	***Bacillaceae***	49	4	13
	***Burkholderiaceae***	3	0	1
	***Intrasporangiaceae***	2	0	1
	***Microbacteriaceae***	9	1	1
	***Micrococcaceae***	48	7	3
	***Mycobacteriaceae***	1	0	1
	***Paenibacillaceae***	7	2	1
	***Planococcaceae***	5	0	0
	***Pseudonocardiaceae***	2	0	0
	***Staphylococcaceae***	11	1	5
	***Streptomycetaceae***	4	0	1
**Total**	**13**			
**Phyla**	***Actinobacteria***	66	8	7
	***Firmicutes***	73	7	19
	***Proteobacteria***	4	0	2
**Total**	**3**	**143**	**15**	**28**

## 2 Materials and methods

### 2.1 Isolation, molecular identification and phylogenetic analysis of bacterial isolates

Soil samples were collected in May 2015 from maize rhizospheres at a farm in the Ngaoundal locality. The isolation of microorganisms was assessed in non-selective nutrient agar (NA) medium (Standard nutrient agar I, Carl Roth, Germany) containing 6 g NaCl, 3 g yeast extract, 15 g peptone, 1 g glucose, 12 g agar-agar L^−1^, pH 7. Bacterial colonies were selected based on their morphological characteristics [4] and purified. Genomic DNA was extracted from overnight pure bacterial culture grown in nutrient broth (Standard nutrient broth I, Carl Roth, Germany) at 28 °C was performed using the DNeasy Plant Mini kit (QIAGEN, Germany) by following the manufacturer׳s instructions. The genomic DNA extracted from all isolates was used for partial 16S rRNA gene amplification using two 16S rDNA sequencing universal primers: 9bfm (5′ -GAGTTTGATYHTGGCTCAG-3′) and 1512R (5′ -ACGGHTACCTTGTTACGACTT-3′) [5]. All PCR amplicons were confirmed by electrophoresis, purified and sequenced. The bacterial 16S rDNA nucleotide sequences were aligned with known sequences in the NCBI (http://blast.ncbi.nlm.nih.gov) and Ribosomal Database Project (RDP) databases using BLASTn. Multiple sequence alignments with the most closely related bacterial sequences were performed using Muscle (https://www.ebi.ac.uk/Tools/msa/muscle/) and phylogeny was inferred by the Maximum Likelihood approach based on the Tamura 3-parameter model and the neighbor-joining method [6], using Mega 7 version 7.0.21 (http://www.megasoftware.net/).

### 2.2 *In vitro* characterization of bacterial isolates

All identified bacterial strains were characterized *in vitro* by the salt tolerance, phosphate solubilization, *nifH* gene presence and siderophore production capacity. The salinity tolerance potential was evaluated by observing the growth on Standard I Nutrient agar (Carl Roth, Germany) amended with various concentrations of NaCl (2%, 4%, 6%, and 8% w-v) [7]. The ability of isolates to solubilize seven different inorganic phosphate sources (tricalcium phosphate, hydroxyapatite, Malian rock phosphate (RP), Cameroonian RP, Algerian RP, Mexican RP, Moroccan RP) was assessed on plates filled with the National Botanical Research Institute׳s Phosphate growth medium (NBRIP) [8]. Potential nitrogen-fixing bacteria were determined by searching for the presence of the *nifH* gene, the marker gene for biological nitrogen fixing ability using the universal primers 19F (5′-GCIWTYTAYGGIAARGGIGG-3′) and 366R (5′-AAICCRCCRCAIACIACRTC-3′) [9]. Siderophore production by bacterial isolates was determined following the universal assay of Schwyn and Neilands using CAS-blue plates [10].
