# Supplementary figures and images for: Data on molecular identification, phylogeny and in vitro characterization of bacteria isolated from maize rhizosphere in Cameroon
Source: Data Brief. 2018 Jun 11;19:1410–7. doi: 10.1016/j.dib.2018.06.003 (PMC6141133; doi:10.1016/j.dib.2018.06.003)

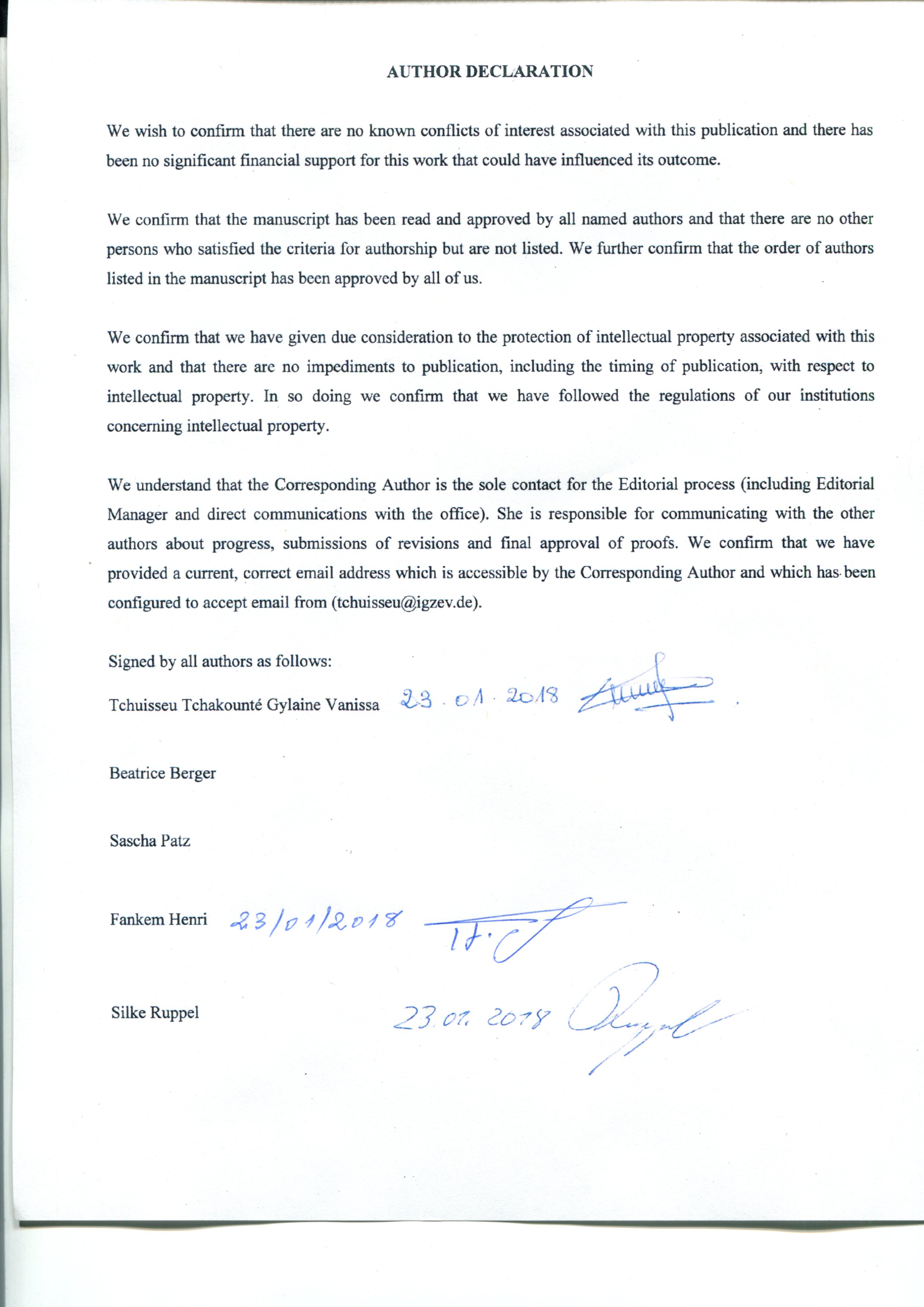

Supplement: Supplementary file 1 — Transparency document [file mmc1.zip › mmc1/Author declaration 2.jpg]
